# PFunkel: Efficient, Expansive, User-Defined Mutagenesis

**DOI:** 10.1371/journal.pone.0052031

**Published:** 2012-12-17

**Authors:** Elad Firnberg, Marc Ostermeier

**Affiliations:** Department of Chemical and Biomolecular Engineering, Johns Hopkins University, Baltimore, Maryland, United States of America; Cardiff University, United Kingdom

## Abstract

We introduce PFunkel, a versatile method for extensive, researcher-defined DNA mutagenesis using a ssDNA or dsDNA template. Once the template DNA is prepared, the method can be completed in a single day in a single tube, and requires no intermediate DNA purification or sub-cloning. PFunkel can be used for site-directed mutagenesis at an efficiency approaching 100%. More importantly, PFunkel allows researchers the unparalleled ability to efficiently construct user-defined libraries. We demonstrate the creation of a library with site-saturation at four distal sites simultaneously at 70% efficiency. We also employ PFunkel to create a comprehensive codon mutagenesis library of the *TEM-1* ß-lactamase gene. We designed this library to contain 18,081 members, one for each possible codon substitution in the gene (287 positions in *TEM-1* x 63 possible codon substitutions). Deep sequencing revealed that ∼97% of the designed single codon substitutions are present in the library. From such a library we identified 18 previously unreported adaptive mutations that each confer resistance to the ß-lactamase inhibitor tazobactam. Three of these mutations confer resistance equal to or higher than that of the most resistant reported *TEM-1* allele and have the potential to emerge clinically.

## Introduction

An efficient and high-throughput mutagenesis strategy is an integral part of protein structure/function studies, directed evolution experiments for the discovery of novel proteins, and optimization of genetic elements in synthetic biology systems. Among the methods for in vitro mutagenesis, none offers a convenient, efficient and high-throughput approach for creating an extensive, user-defined library of variants in which single or multiple mutations can be located at any position. Site-directed mutagenesis methods such as Kunkel mutagenesis [1], QuikChange [2], and inverse PCR [3] are low-throughput methods. Combined chain reaction requires specially designed sets of primers and cloning of PCR products [4], [5]. Creating mutations by gene synthesis is comparatively expensive and requires sub-cloning of DNA. Error-prone PCR suffers from mutational bias, the inability to define the mutational composition, and the inability to effectively cause most amino acid substitutions, which require two or three mutations in a single codon. Methods that rely on random DNA cleavage reagents or transposons for mutating short sequences of DNA suffer from complex procedures and the inability to target the mutations [6]–[8].

Our method is inspired by Kunkel mutagenesis, a site-directed method that introduces mutations by using a mutation-encoding oligonucleotide (oligo) that anneals to a phage-derived, single-stranded uracil-containing circular DNA template. While the initial Kunkel protocol described making single base substitutions [1], other researchers have adapted the method for creating site-saturation libraries in a single region [9], [10]. The mutational efficiency of Kunkel mutagenesis is limited such that 50–90% of transformed colonies typically harbor the desired mutation while the remainder harbor the wildtype sequence [11].

PFunkel, a conflation of *Pfu* DNA polymerase and Kunkel mutagenesis and pronounced “pee-funk-el”, differs from Kunkel mutagenesis in a number of key ways that serve to increase the efficiency of the reaction and minimize the appearance of wildtype sequences in the resulting library. The major differences include (a) the use of a thermostable DNA polymerase and ligase, which enables a shift in the operating temperature of the reaction from 25–37°C to 55–95°C, (b) the option to use thermal cycling and stepwise addition of oligos to tailor the average number of mutations per gene, (c) the synthesis of a second mutated strand complementary to the first mutated strand that displaces the template strand, and (d) the in vitro degradation of the uracil-containing template and DNA products not in the desired covalently closed circular (cccDNA) form by the addition of uracil DNA glycosylase (UDG) and exonuclease III (Exo III). Additionally, we have developed a version of PFunkel that can be performed on any dsDNA plasmid template and avoids the use of phage.

We demonstrate PFunkel on the *TEM-1* gene encoding TEM-1 ß-lactamase by performing three types of mutagenesis experiments: (a) site-directed mutagenesis with 100% efficiency, (b) multiple-site mutagenesis, in which we create site-saturation libraries at four distal codons in *TEM-1* at ∼70% efficiency, and (c) a new type of mutagenesis library called comprehensive codon mutagenesis. A comprehensive codon mutagenesis library consists of every possible codon substitution in the gene with only one codon substitution per library member (i.e. library members containing more than one codon mutated are not desired). Such a library is the equivalent of creating a site-saturation mutagenesis library at all positions in the gene. The degeneracy of this library for *TEM-1* is 18,081 (287 codons × 63 possible codon substitutions). Deep sequencing of our library revealed that up to 97% of the possible 18,081 possible desired codon substitutions exist in the library and that the fraction of wildtype and variants with two or more codon substitutions in the library was ∼13% and <3%, respectively.

**Figure 1:**
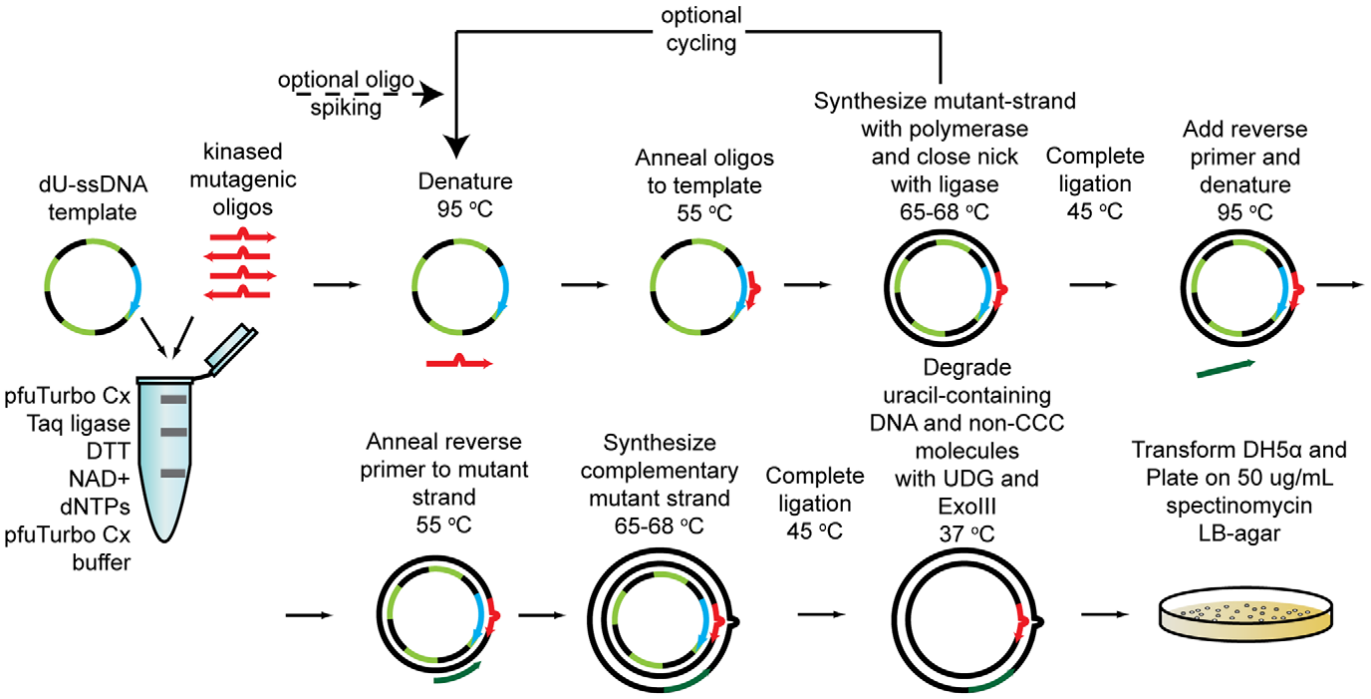
Schematic of PFunkel mutagenesis using a ssDNA template. The basic protocol is depicted. For multiple-site mutagenesis, the addition of the polymerase, dNTPs, ligase, DTT, and NAD^+^ is delayed until after the first annealing step. For comprehensive codon mutagenesis, the ratio of oligo to template is kept low to minimize multiple mutations in a single reaction product. Cycling with occasional spiking of additional mutagenic oligos improves the reaction yield.

## Materials and Methods

All enzymes were obtained from New England Biolabs (NEB) except PfuTurbo Cx hotstart DNA polymerase, which was obtained from Agilent Technologies. *E. coli* strain CJ236 and NEB 5-alpha F’I^q^ competent cells were obtained from NEB and strain DH5α was obtained from Invitrogen. R408 helper phage was obtained from Promega. All oligonucleotides were ordered from Integrated DNA Technologies. For the construction of library CCM-1 (machine-mixed degenerate oligos), oligos were ordered in 96-well format at the 10 nmole synthesis scale such that each oligo was provided at a concentration of 100 µM in DI water. For the construction of library CCM-2 (hand–mixed degenerate oligos), oligos were ordered in 96-well format at the 100 nmole synthesis scale such that each oligo was provided at a concentration of 100 µM in DI water. The secondary oligo P320, P-gcagaaattcgaaagcaaattcgac, was ordered with 5′ phosphorylation. All other chemical reagents were obtained from Sigma-Aldrich.

**Table 1:** Statistics of comprehensive codon mutagenesis library CCM-1.

	Expected in an ideal library	Sequencing of individual clones of the library	Sequencing of PCR amplicons used in 454 sequencing	454 sequencing of the library	454 sequencing of *TEM-1*
Sequences		90 clones	28 clones	787,488 reads	2040 reads
Percent of reads that cover entire gene segment		100%	100%	98.95%	99.75%
Number of mutated codons in all sequences		78+2^a^	22	738,615	72
Mean mutated codons per sequence	0.9844	0.87	0.79	0.94	0.035
**Percent of clones/reads with**					
No mutations	1.56%	13.33%	35.71%	26.17%^b^	96.9%
One mutation	98.44%	86.67%	50.00	56.71%	2.75%
Multiple mutations	0.00%	0.00%	14.29%	17.12%^b^	0.034%
**Percent of mutated codons with**					
1 base substitution	14.29%	22.50%	22.72%	31.97%	86.11%
2 base substitution	42.86%	47.50%	59.10%	41.84%	13.89%
3 base substitution	42.86%	30.00%	18.18%	26.20%	0.00%
**Percent of possible codon substitutions observed**					
1 base substitution				99.96%	
2 base substitutions				97.70%	
3 base substitutions				95.33%	
All substitutions				97.01%	

a two mutations were identified outside the region targeted for mutagenesis.

b most of the reads with multiple mutations and about 50% of the reads with no mutations result from PCR jumping during amplicon creation (see Text S1). The library mostly is comprised of members with one mutation as indicated in sequencing of individual clones.

### Preparation of CJ236 Competent Cells

*E. coli* strain CJ236 was plated on LB-agar plates with 15 µg/mL chloramphenicol (Cm), 125 µg/mL deoxythymidine (dThd) and grown at 30°C. Although it is usual to proceed with competent cell preparation from a single colony (especially if using a new cell stock validated by the manufacturer), we chose to first confirm the desired strain phenotype, an optional step. A colony with the proper temperature-sensitive *dut-1* phenotype was identified by replica plating on M9 minimal media agar [12] supplemented with and without 125 µg/mL dThd and incubated at 30°C and 42°C for ∼40 hours. A colony was selected which displayed the desired phenotype of stunted growth at 42°C, which was improved with dThd (Personal correspondence with B. Weiss). This colony was used to prepare chemically competent cells [13]. To prevent genetic drift and reversal of the *dut-1 ung-1* phenotype it is best to propagate CJ236 at ≤30°C in dThd supplemented media. These conditions reduce uracil incorporation in DNA (an unfavorable mutagenic event leading to reversions of this phenotype) since uracil incorporation is unnecessary when propagating the strain. However, during preparation of uracil-containing ssDNA or dsDNA template, the strain should be grown at 37°C without dThd for increased uracil incorporation.

**Figure 2:**
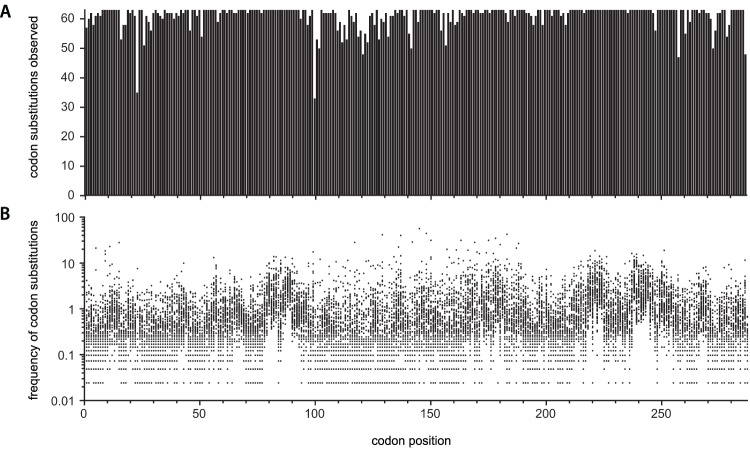
Completeness and frequency of codon substitutions observed in 454 sequencing of the comprehensive codon mutagenesis library of *TEM-1*. (**a**) Number of the 63 possible codon substitutions observed and (**b**) frequency of codon substitutions observed as a function of position in the gene. For each of the 287 codons of *TEM-1* the frequency of each of the 63 possible codon substitutions is shown, except for the 3% of the 18,081 codon substitutions that were not observed. The frequency is based on 454 sequencing in which 738,615 codon substitutions were observed in 787,488 reads. The frequency is normalized to the frequency that would occur if all substitutions were evenly distributed among the 18,081 possible substitutions (i.e. frequency = 1.0 means that the substitution was observed 738,615/18,081 = 41 times). The number of codon substitutions observed resulting from sequencing errors is small (∼4% of the 738,615 codon substitutions observed).

### Preparation of Uracil-containing ssDNA Template

pSkunk3-BLA is a 4.4 kB phagemid derived from pDIM-C8-BLA [14] in which the coding sequence of the Cm resistance gene was replaced with the streptomycin/spectinomycin (Sm/Spec) resistance gene. This phagemid was used to transform CJ236 cells which were then plated on LB-agar with 50 µg/mL Spec, 15 µg/mL Cm, and 125 µg/mL dThd and incubated overnight at 30°C. A single colony was used to inoculate 10 ml of LB supplemented with Cm, Spec, and dThd as above, which was incubated with shaking at 30°C overnight. The cell density of the culture was determined from the OD_600nm_ using the correlation 2×10^8^ CFU/mL-OD_600nm_. In a 20 ml test tube, 2 ml of TBG media [15] with 50 µg/mL Spec was inoculated with 2×10^7^ CFU from the overnight culture and 1×10^8^ pfu R408 helper phage for an MOI of 5. This culture was incubated for 6 hours at 37°C with shaking at 300 rpm. The culture was then centrifuged for 5 minutes at 16,100×g to pellet the cells, and the phage-containing supernatant recovered. Then 300 µL of 2.5 M NaCl/20% PEG was added to the supernatant and the mixture was incubated at 4°C for 1 hour to precipitate the phage. The phage was pelleted by centrifugation at 20,817×g for 10 minutes at 4°C. The liquid supernatant was discarded and the phage pellet resuspended in 150 µL PBS. The Qiagen QIAprep Spin M13 kit (#27704) was then used to purify ssDNA from the phage as per the manufacturer’s directions. The absorbance at 260 nm of the ssDNA sample was measured using a Nanodrop ND-1000 spectrophotometer (Thermo Scientific) and converted to a concentration using the relation 1.0 A_260nm_ = 33 ng/µL.

**Table 2:** Potential adaptive amino acid substitutions in *TEM-1* identified from genetic selections for tazobactam resistance codon substitutions.

Ambler position^a^	Amino acid substitutions clinically observed^b^	Amino acid substitutions observed in this study^c^	Occurrences^d^	Codon coverage^e^
I13	–	**L**	2	2 of 6
L21	F, I	Q	2	1 of 2
M69	L, I, V	**L**	30	6 of 6
Q90	–	A	2	1 of 4
Y105	–	G	14	4 of 4
		S*	10	3 of 6
		A	7	2 of 4
		**D**	5	2 of 2
		**N**	5	2 of 2
		W	4	1 of 1
		T	2	2 of 4
R120	G	E	3	1 of 2
S124	N	Q	2	1 of 2
T128	–	E	2	1 of 2
T140	–	G	2	1 of 4
E147	–	**G**	2	2 of 4
W165	R, C, G	Y	4	2 of 2
S235	–	**T**	8	3 of 4
T265	M	**M**	4	1 of 1

a Numbering according to Ambler et al. [28].

b Amino acid substitutions observed in natural alleles of *TEM-1* with increased resistance to b-lactam antibiotics or ß-lactamase inhibitors (http://www.lahey.org/studies/temtable.asp). Amino acid substitutions underlined are found in alleles with increased inhibitor resistance [29].

c Amino acid substitutions in bold were observed with a single base change in the codon. * means that although the amino acid substitution can occur with a single base change, such a change was not observed here.

d Of the amino acid substitution in this study.

e For the amino acid substitutions found in this study, the number of unique codons observed out of the possible number of unique codons is reported.

### Site-directed PFunkel Mutagenesis using a ssDNA Template

All steps were performed in a pre-programmed Eppendorf Mastercycler personal thermocycler. A mutagenic oligo (5′-gacaccacgatgcatgcagcaatggc) encoding a c542a mutation in the *bla* gene was phosphorylated in a 50 µL reaction containing 1X T4 PNK buffer, 1 mM ATP, 5 mM DTT, 3.0 uM oligo and 10 units T4 PNK. The reaction was incubated at 37°C for 1 hour, and the enzyme inactivated at 65°C for 20 minutes.

**Figure 3:**
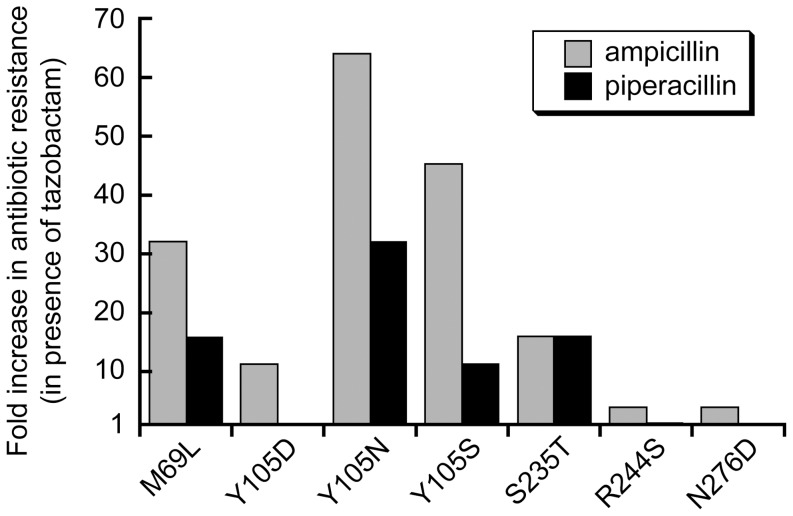
Tazobactam resistance of selected alleles. The increase in ampicillin or piperacillin resistance is reported as the fold increase (over *TEM-1*) in the minimum inhibitory concentration (MIC) of the antibiotic in the presence of 6 µg/ml tazobactam. MIC assays performed in √2-fold increments of antibiotic concentration. Median MIC values of three replicates were used. Data for all replicates is in Tables S6 and S7.

The PFunkel reaction was prepared in a 0.5 mL eppendorf tube containing 1X PfuTurbo Cx hotstart DNA polymerase buffer, 10 mM DTT, 0.5 mM NAD+, 0.2 mM dNTPs, 1 µL of the kinase reaction, 1 µg (0.75 pmol) of dU-ssDNA template, 2.5 units PfuTurbo Cx hotstart DNA polymerase, and 200 cohesive end units Taq ligase in a total volume of 100 µL. The free Mg^2+^ concentration should be maintained between 0.5–2.5 mM since low concentration reduces polymerase fidelity while high concentration leads to nonspecific annealing of oligos [16]. The volume of kinase reaction added should therefore be minimized to maintain Mg^2+^ concentration in the mutagenesis reaction close to the 2 mM Mg^2+^ provided in the 1X polymerase buffer. The following denaturation/annealing/extension/ligation steps were performed: 95°C for 3 min, 55°C for 90 sec, 68°C for 15 min and 45°C for 15 min. Then 3.8 pmol of oligo P320 (5′-P-gcagaaattcgaaagcaaattcgac) was added and one more cycle of 95°C for 30 sec, 55°C for 45 sec, 68°C for 10 min and 45°C for 15 min was performed. Then 10 units of UDG and 30 units of ExoIII were added and incubated at 37°C for 1 hr followed by an inactivation step at 70°C for 20 min.

Five µL of the unpurified reaction was used to directly transform 100 µL of DH5α chemically competent cells [13]. The entire transformation was plated on an LB-agar plate with 50 µg/mL Spec and incubated overnight at 37°C. To obtain more transformants, the remaining DNA was purified using the Zymo DNA Clean & Concentrator kit according to the manufacturer’s instructions and eluted in 15 µL of 1X EB. One uL was electroporated into 50 µL DH5α electrocompetent cells and then incubated with SOC recovery media for 1 hr at 37°C with shaking at 250 rpm. The transformation was plated on LB-agar with 50 µg/mL Spec and incubated overnight at 37°C.

For the experiments of Table S2, the reaction was scaled down to 200 ng template and 20 µl volume.

### Multi-site PFunkel Mutagenesis using a ssDNA Template

Four oligos were designed to introduce NNN random bases at codon positions 42, 104, 182, and 238 in the *bla* gene with respective sequences: 5′-gatcagttgggtnnncgagtgggttac, 5′-gaatgacttggttnnntactcaccagtcac, 5′-cgtgacaccacgnnncctgcagcaatg, 5′-aaatctggagccnnngagcgtgggtct. These oligos were combined in equimolar amounts and phosphorylated in a 50 µL reaction containing 1X T4 PNK buffer, 1 mM ATP, 5 mM DTT, 6.0 µM total oligo and 10 units T4 PNK. The reaction was incubated at 37°C for 1 hour, and the enzyme inactivated at 65°C for 20 minutes.

The annealing reaction was prepared in a 0.5 mL eppendorf tube containing 1X PfuTurbo Cx hotstart DNA polymerase buffer, 2 uL of kinase reaction, and 1 ug of pSkunk3-bla ssDNA template in a total volume of 77 uL. The annealing was performed by heating to 95°C for 3 min, then 55°C for 10 min, and holding at 55°C.

Meanwhile, in a separate PCR tube, 1X PfuTurbo Cx hotstart DNA polymerase buffer and 2.75 units of PfuTurbo Cx hotstart DNA polymerase were combined in a total volume of 5.5 µL. The hotstart polymerase was heat activated by heating to 95°C for 3 min.

After the annealing step, 10 mM DTT, 0.5 mM NAD+, 0.2 mM dNTPs, 5 µL of the activated polymerase solution, and 200 cohesive end units Taq ligase was added bringing the total volume to 100 µL. The reaction was mixed by slowly and gently pipetting up and down. Extension and ligation of the mutant strand was performed at 65°C for 15 min and 45°C for 15 min. A total of 3.8 pmol of oligo P320 was added and one more cycle of 95°C for 30 sec, 55°C for 45 sec, 65°C for 10 min and 45°C for 15 min was performed. Five units of UDG and 2 units of ExoIII were added and the mixture was incubated at 37°C for 30 min followed by an inactivation step at 70°C for 20 min. The DNA was then purified using the Zymo DNA Clean & Concentrator kit according to the manufacturer’s instructions and eluted in 15 µL of DI water. This volume was vacuum concentrated down to 1–2 µL, electroporated into 50 µL DH5α electrocompetent cells and then incubated with SOC recovery media for 1 hr at 37°C with shaking at 250 rpm. The entire volume was then plated on a Nalgene Bioassay dish (D4803; 245 mm×245 mm×25 mm) containing LB-agar with 50 µg/mL Spec and incubated overnight at 37°C.

For the experiments of Table S2, the reaction was scaled down to 200 ng template and 20 µl volume.

### Comprehensive Codon Mutagenesis by PFunkel using a ssDNA Template

All steps were performed in a pre-programmed Eppendorf Mastercycler personal thermocycler. Equimolar amounts of 287 different mutagenic oligos were combined in a single tube at a total oligo concentration of 100 µM. The oligos were phosphorylated in a 50 µL reaction containing 1X T4 PNK buffer, 1 mM ATP, 5 mM DTT, 0.038 µM oligos and 10 units T4 PNK. The reaction was incubated at 37°C for 1 hour, and the enzyme inactivated at 65°C for 20 minutes.

The PFunkel reaction was prepared in a 0.5 mL eppendorf tube containing 1X PfuTurbo Cx hotstart DNA polymerase buffer, 10 mM DTT, 0.5 mM NAD+, 0.2 mM dNTPs, 1 µL of the kinase reaction, 1 µg (0.75 pmol) of dU-ssDNA template, 2.5 units PfuTurbo Cx hotstart DNA polymerase, and 200 cohesive end units Taq ligase in a total volume of 100 µL. The following denaturation/annealing/extension steps were performed: 95°C for 2 min, 15 cycles of 95°C for 30 sec, 55°C for 45 sec, and 68°C for 6.5 min. At the 95°C step of cycles 6 and 11, 1 µL of the kinase reaction was added and mixed in by stirring with the pipette tip. The reaction was then incubated at 45°C for 15 min for ligation to occur. Then 3.8 pmol of oligo P320 (5∶1 molar ratio oligo to template) was added and one more cycle of 95°C for 30 sec, 55°C for 45 sec, and 68°C for 10 min was carried out. The reaction was again incubated at 45°C for 15 min. Then 10 units of UDG and 30 units of ExoIII were added and incubated at 37°C for 1 hr followed by an inactivation step at 70°C for 20 min. The DNA was then purified using the Zymo DNA Clean & Concentrator kit according to the manufacturer’s instructions and eluted in 15 µL of DI water. This volume was then vacuum concentrated down to 1–2 µL. For CCM-1 the DNA was electroporated into 50 µL DH5α electrocompetent cells and then incubated with SOC recovery media for 1 hr at 37°C with shaking at 250 rpm. The entire volume was then plated on a Nalgene Bioassay dish (D4803; 245 mm×245 mm×25 mm) containing LB-agar with 50 µg/mL Spec and incubated overnight at 37°C. For CCM-2, the DNA was used to transform NEB 5-alpha F’I^q^ competent cells as per the manufacturer’s instructions, and then plated on a Nalgene Bioassay dish containing LB-agar with 50 µg/mL Spec, 15 µg/mL tetracycline, and 2 w/v% glucose.

### 454 GS FLX High-throughput Sequencing

Transformants were recovered from agar plates with LB broth, and plasmid DNA recovered using the Qiagen QIAprep Spin Miniprep kit (27106). The plasmid DNA was linearized by restriction endonuclease digestion with NdeI. PCR amplicons of each of the three *bla* libraries were created using Titanium Lib-A fusion primers that included a 10-base MID barcode. Each 25 µL PCR reaction had 1–2 ng linearized template DNA, 0.4 µM each primer, 200 µM each dNTP, 1X HF Phusion buffer, and 2 units Phusion high-fidelity polymerase. Cycler conditions were 98°C for 30 sec, 30 cycles of 98°C for 30 sec, 55°C for 30 sec, 72°C for 30 sec, and then 72°C for 5 min. PCR products were visualized on an ethidium bromide 1% agarose gel, and then gel purified using the QIAquick Gel Extraction Kit (28706). Amplicons were furthered purified using the Agencourt AMPure XP PCR Purification kit (A63880), to remove short DNA fragments, primers, and primer dimers. DNA concentration was determined using the Quant-iT Picogreen dsDNA Assay kit (P7589). Amplicons from each sub-library were diluted to 1E9 molecules/µL in 1X TE, equal volumes pooled together and then further diluted to 1E7 molecules/µL in DI water. 454 sequencing was performed by Tufts University Core Facility on a Roche 454 GS FLX+ instrument. The sequencing data was then analyzed using the Galaxy open web-based platform [17]–[19] and custom Matlab scripts.

### Identification of Adaptive Codon Substitutions for Tazobactam Resistance in *TEM-1*

Library CCM-2 was plated at a density of about 500 CFU/cm^2^ (non-selective conditions) on LB-agar plates supplemented with 50 µg/ml Spec, 300 µM IPTG, 100 µg/ml ampicillin and 4 or 6 µg/ml tazobactam. Plates were incubated at 37°C for 17 hours. The tazobactam concentration chosen was 1.3 or 2-fold higher than the concentration at which cells bearing wildtype *TEM-1* could grow effectively. Large colonies on the plates were chosen at random for sequencing.

Selected single base mutations were re-introduced into *TEM-1* by site-directed PFunkel mutagenesis on the 20 µl volume scale. The MIC for ampicillin and piperacillin of the mutants was assessed with and without 6 µg/ml tazobactam by spotting 10^4 ^CFU on Mueller-Hinton agar plates containing 50 µg/ml Spec, 300 µM IPTG, and √2-fold increments of either ampicillin or piperacillin. Plates were incubated at 37°C for 12 hrs.

### Preparation of Uracil-containing dsDNA Template for Phage-less PFunkel

A 10 mL LB culture of CJ236 cells with the pSkunk3-bla plasmid was incubated overnight at 37°C with shaking at 250 rpm. Plasmid dU-dsDNA was then isolated using the Qiagen QIAprep Spin Miniprep kit (27106) and the concentration quantified using a Nanodrop ND-1000 spectrophotometer.

### Site-directed PFunkel Mutagenesis using a Plasmid dsDNA Template

All steps were performed in a pre-programmed Eppendorf Mastercycler personal thermocycler. A mutagenic oligo (5′-gacaccacgatgcatgcagcaatggc) encoding a c542a mutation in *TEM-1* was phosphorylated in a 50 µL reaction containing 1X T4 PNK buffer, 1 mM ATP, 5 mM DTT, 1.5 µM oligo and 10 units T4 PNK. The reaction was incubated at 37°C for 1 hour and the enzyme inactivated at 65°C for 20 minutes.

The PFunkel reaction was prepared in a 0.5 mL tube containing 1X PfuTurbo Cx hotstart DNA polymerase buffer, 10 mM DTT, 0.5 mM NAD+, 0.2 mM dNTPs, 1 µL of the kinase reaction, 1 µg (0.38 pmol) of dU-dsDNA template, 2.5 units PfuTurbo Cx hotstart DNA polymerase, and 200 cohesive end units Taq ligase in a total volume of 100 µL. The free Mg^2+^ concentration should be maintained between 0.5–2.5 mM since low concentration reduces polymerase fidelity while high concentration leads to nonspecific annealing of oligos [16]. The volume of kinase reaction added should therefore be minimized to maintain Mg^2+^ concentration in the mutagenesis reaction close to the 2 mM Mg^2+^ provided in the 1X polymerase buffer. The following denaturation/annealing/extension/ligation steps were performed: 95°C for 3 min, 55°C for 90 sec, 68°C for 15 min and 45°C for 15 min. Next, 10 units of UDG and 30 units of Exo III were added and the reaction was incubated at 37°C for 1 hr followed by an inactivation step at 70°C for 20 min. A total of 3.8 pmol of oligo P320 (5′-P-gcagaaattcgaaagcaaattcgac) was added and one more cycle of 95°C for 30 sec, 55°C for 45 sec, 68°C for 10 min and 45°C for 15 min was performed. The DNA was purified using the Zymo DNA Clean & Concentrator kit according to the manufacturer’s instructions and eluted in 15 µL of DI water. This solution was vacuum concentrated down to 1–2 µL, electroporated into 50 µL DH5α electrocompetent cells, which were incubated with SOC recovery media for 1 hr at 37°C with shaking at 250 rpm. The transformation was plated on LB-agar with 50 µg/mL Spec and incubated overnight at 37°C.

### Multi-site PFunkel Mutagenesis using a Plasmid dsDNA Template

Four oligos were designed to introduce the four mutations A42G, E104K, M182T, and G238S in the *bla* gene with respective sequences: 5′-gatcagttgggtgga cgagtgggttac, 5′-ctcagaatgacttggttaagtactcaccagtcacag, 5′-gtgacaccacgacgcctgcagcaatggcaacaac, 5′-gctgataaatctggagccagtgagcgtgggtctcg. These oligos were combined in equimolar amounts and phosphorylated in a 50 µL reaction containing 1X T4 PNK buffer, 1 mM ATP, 5 mM DTT, 3 µM total oligo and 10 units T4 PNK. The reaction was incubated at 37°C for 1 hour and the enzyme inactivated at 65°C for 20 minutes.

The annealing reaction was prepared in a 0.5 mL eppendorf tube containing 1X PfuTurbo Cx hotstart DNA polymerase buffer, 2 µL of kinase reaction, and 1 µg of dU-dsDNA template in a total volume of 77 µL. The annealing was performed by heating to 95°C for 3 min, then 55°C for 10 min, and holding at 55°C.

Meanwhile, in a separate PCR tube, 1X PfuTurbo Cx hotstart DNA polymerase buffer and 2.75 units of PfuTurbo Cx hotstart DNA polymerase polymerase were combined in a total volume of 5.5 µL. The hotstart polymerase was heat activated by heating to 95°C for 3 min.

After the annealing step, 10 mM DTT, 0.5 mM NAD+, 0.2 mM dNTPs, 5 µL of the activated polymerase solution, and 200 cohesive end units Taq ligase was added bringing the total volume to 100 µL. The reaction was mixed by slowly and gently pipetting up and down. Extension and ligation of the mutant strand was performed at 65°C for 15 min and 45°C for 15 min. Five units of UDG and 2 units of ExoIII were added and the mixture was incubated at 37°C for 1 hr followed by an inactivation step at 70°C for 20 min. A total of 3.8 pmol of oligo P320 was added and one more cycle of 95°C for 30 sec, 55°C for 45 sec, 68°C for 10 min and 45°C for 15 min was performed. The DNA was purified using the Zymo DNA Clean & Concentrator kit according to the manufacturer’s instructions and eluted in 15 µL of DI water. This solution was vacuum concentrated down to 1–2 µL, electroporated into 50 µL DH5α electrocompetent cells and then incubated with SOC recovery media for 1 hr at 37°C with shaking at 250 rpm. The transformation was plated on LB-agar with 50 µg/mL Spec and incubated overnight at 37°C.

## Results

### Limitations of Kunkel Mutagenesis

We sought to minimize the occurrence of wild-type sequences in Kunkel mutagenesis, which has been reported to be as high as 30–50% [11]. We postulated that wild-type sequences arise for several reasons. First, the low operating temperature of the second strand synthesis step allows any contaminating short DNA fragments in the single stranded DNA prep, termed “junk” DNA, to prime the single stranded DNA. Such synthesis can either create a wild-type double stranded product or poison a mutation-bearing product by creating reaction side-products that possess a nick or a displaced strand [20]. Additionally, at lower temperatures, the mutagenic oligos are more prone to anneal non-specifically to the template. Such reaction side-products that are not in the cccDNA form are prone to degradation by cellular nucleases, removing the mutation. The presence of junk DNA is apparent from DNA gels of reaction products in which no mutagenic oligonucleotides were added, yet higher molecular weight products are produced [21]. Another postulated reason for the high occurrence of wild-type sequences in Kunkel mutagenesis is the repair of the mutation by mismatch-repair machinery or repair of the uracil-containing template strand in the cell after transformation.

### Overview of PFunkel Mutagenesis using a ssDNA Template

The in vitro reaction steps of PFunkel mutagenesis (Figure 1, Table S1) are designed to eliminate products other than the desired mutated cccDNA plasmid molecules, resulting in high mutational efficiencies. Other than the initial kinase reaction to phosphorylate the mutagenic oligonucleotides, all reaction steps are conveniently performed in the same tube with no DNA purification required except as an optional final step to improve transformation efficiency. PFunkel is conveniently performed in a thermocycler.

Uracil-containing ssDNA is produced by propagating phagemid DNA containing the DNA to be mutated in an *E. coli dut-1* *ung-1 *host, then infecting the culture with M13 helper phage and harvesting ssDNA from the resulting phage particles. *E. coli dut-1* *ung-1* strains express a heat-sensitive dUTPase that has 5% of wildtype activity at 25°C but <1% at 37°C, and are deficient in uracil DNA glycosylase activity [22]. The result of these mutations is the accumulation of high levels of intracellular dUTP that becomes incorporated in DNA in place of dTTP during DNA synthesis and is not removed due to the lack of UDG activity. ssDNA preparation takes only a day and requires no special laboratory equipment or highly specialized training [21].

To minimize second strand synthesis originating from junk DNA annealing to the uracil-containing ssDNA template, we shifted the operating temperature from the 25–37°C to 55–95°C. This operating temperature required a high-fidelity thermostable polymerase capable of using a uracil-containing template. Additionally, a polymerase lacking strand displacement activity would be advantageous for creating multiple mutations simultaneously at different sites in a gene. The only commercially available polymerase that met these criteria was PfuTurbo Cx hotstart DNA polymerase (Agilent), a variant of *Pfu* polymerase with the V93Q mutation [23]. This mutation inactivates the uracil-binding pocket of the enzyme that would normally cause it to stall at uracil bases. At ≤68°C PfuTurbo Cx hotstart DNA polymerase does not strand-displace but still maintains significant polymerase activity [24]. Taq ligase was chosen due to its effectiveness in ligating DNA nicks, robust activity from 45°C to 65°C, and ability to withstand many rounds of temperature cycling.

In order to create the dsDNA product with the designed mutation on both strands, an excess of a ‘reverse’ oligonucleotide that anneals outside of the gene on the newly created mutant strand is added, such that it primes synthesis of a new complementary strand that encodes the desired mutations and displaces the uracil-containing template. Treatment with UDG acts to excise the uracil bases from the original template strand leaving apyrimidinic (AP) sites. Treatment with exonuclease III (ExoIII), which has both AP-site endonuclease and 3′->5′ exonuclease activity [25], acts to create nicks at the AP sites and then digests the template strand at the nicks and from any 3′ end in the context of dsDNA.

### Site-directed PFunkel Mutagenesis

A mutagenic oligonucleotide encoding a c542a (P183H in the protein) mutation in the TEM-1 ß-lactamase (*TEM-1*) gene was first 5′ phosphorylated in a kinase reaction. The phosphorylated oligo was then combined with the ssDNA uracil-containing template in molar ratio of 4∶1 together with the polymerase and ligase. The incubation temperatures were cycled to perform a denaturing, annealing, extension, and ligation step to complete the mutated second strand and seal the nick. A second primer that annealed to the new strand outside the gene was added to the reaction, and the denaturing, annealing, extension, and ligation steps were repeated. Exo III and UDG were then added to the reaction to remove the template and undesired side-products. All steps for this procedure took about 3 hrs to complete.

A transformation of 5 µL of the unpurified reaction with 100 µL of chemically competent cells yielded over 1000 transformants, illustrating that DNA purification is not necessary. The remaining DNA was purified using a spin column and 1/15^th^ of the product was electroporated into electrocompetent DH5α *E. coli* yielding 533,000 transformants. Sequencing of the *TEM-1* gene from 23 colonies showed that all 23 (100%) contained the c542a mutation encoded by the oligo. No undesired mutations were observed. We further substantiated the high mutational efficiency of our method using eleven different oligos encoding either a 1 or 2 base substitution at different locations of the gene (Table S2).

### Multi-site PFunkel Mutagenesis

Existing methods for site-directed mutagenesis at multiple distal sites simultaneously either have complex and multi-step procedures or have not been demonstrated to be efficient enough for library construction [5], [26]. PFunkel was designed in part to allow efficient construction of libraries in which site-saturation mutagenesis (or any user-defined mutational composition) can be performed at multiple sites simultaneously in a single reaction.

For simultaneous introduction of mutations at multiple distant sites in a gene, the basic PFunkel protocol is modified to increase the frequency of multiple mutations. The polymerase is added after the annealing of the mutagenic oligos. The rationale for the delayed addition of polymerase is to prevent a bias for mutations that result from oligos that anneal efficiently. DNA synthesis from such early annealing oligos might proceed to regions of the gene where other oligos are intended to anneal before the oligos for those locations have a chance to anneal, thus decreasing the frequency of multiple mutations in the resulting transformants. We also reduce the extension temperature to 65°C, to better ensure that PfuTurbo Cx hotstart DNA polymerase does not strand displace. Strand displacement of a strand created from one mutagenic oligo by a strand being synthesized starting from a second mutagenic oligo would reduce the frequency of multiple mutations. We do not know whether the shift of the extension temperature from 68°C to 65°C provides any benefit as we have not performed any direct comparison.

To demonstrate multiple-site mutagenesis using PFunkel, we synthesized four mutagenic oligos designed to create site-saturation libraries of four codons in different regions of the *TEM-1* gene simultaneously. The oligos encoding NNN at codon positions A42, E104, M182, and G238 were combined with the ssDNA template such that each oligo was present in an oligo to template molar ratio of 4∶1. Electroporation of the entire reaction product after spin column purification yielded 5.8 million transformants. Sequencing of the *TEM-1* gene of 10 colonies showed that 7 variants had mutations at all 4 designated codon positions, 2 had mutations at 3 positions, and 1 had mutations at 2 positions (Table S3). Twenty-nine of the 35 codon substitutions were unique and no undesired mutations were observed. We further substantiated multi-site PFunkel by constructing 11 different double, triple, or quadruple mutants at 73% efficiency (Table S2). The error rate for single or multi-site PFunkel mutagenesis was ∼5×10^−5^, higher than expected based on the error rate of PfuTurbo Cx in a PCR reaction (Text S1).

### Comprehensive Codon Mutagenesis

We next used PFunkel to create a library designed to encompass all possible single codon substitutions in the *TEM-1* gene (287 codons x 63 possible codon substitutions at each codon = 18,081 desired mutants). We did not desire library members with more than one codon substituted. Such a library is the equivalent of performing site-saturation mutagenesis at all positions in the gene simultaneously. The advantage of PFunkel is that one does not have to perform 287 separate mutagenesis reactions or 287 separate gene syntheses to create this library. The library would also be much closer to a true random mutagenesis library than one created by error prone PCR, which is biased towards certain base substitutions made by the polymerase and certain amino acid substitutions accomplished by single base mutations.

The 287 degenerate mutagenic oligos (one for each of the 287 codons to be mutated) were designed *in silico* using a Matlab script (Figure S1). The oligos were purchased in desalted 96-well format using machine-mixed degenerate bases and pooled. To minimize the occurrence of multiple mutations, the total oligo to ssDNA template ratio was kept low (1∶20), which makes two oligos annealing to the same ssDNA template unlikely. To increase the yield and efficiency of the reaction, we implemented a cycling reaction of denaturing, annealing, and extension to allow multiple chances for each oligo to productively anneal. Fifteen cycles were performed with additional oligos spiked in at the 6^th^ and 11^th^ cycle. Additional cycles and oligo additions can be performed if larger libraries are desired. Our scheme is analogous to that of a discontinuous fed batch reactor – a reaction strategy to minimize undesirable side products that occur with a high concentration of one of the reactants [27].

Although in principle the library could be created in a single tube, we divided the library into thirds corresponding to each 1/3 of the gene in order to facilitate characterization of the library by 454-GS-FLX Titanium sequencing, which has a read length of ∼400 bp for the sequencing of amplicons from pools of DNA. Transformation of the entire reaction product yielded ∼500,000 transformants for each library. Sequencing of 30 members of each library indicated that the libraries mostly consisted of single codon substitutions (87%) with the remainder being wildtype (13%) (Table 1). No clones with multiple mutations were observed. Two of the sequences contained a single mutation outside the region subjected to mutagenesis, which we attribute to polymerase error. We postulate that additional rounds of cycling and mutagenic oligo addition would lower the fraction of wild-type sequences closer to the theoretical minimum of 1.6% (i.e. 1/64 of the NNN containing oligos encode the wildtype codon). These three libraries collectively were named CCM-1.

We extensively analyzed CCM-1 using 454-GS-FLX Titanium sequencing. Our analysis indicates that 96–97% of the 18,081 desired codon substitutions are present in the library, and ≥97% of library members with a codon substitution contain only one codon substitution (Table 1, Text S1). The frequency of codon substitutions observed as a function of gene position shows that a few positions were hotspots for substitutions and that the frequency of codon substitutions has a broad distribution (Figure 2). Presumably the occurrence of hotspots reflects the suitably of the respective oligos for this mutagenesis technique. Codon substitutions with a single bp change were observed at about twice the expected frequency, and this comes at the expense of fewer codon substitutions with three bp changes (Table 1). A portion of the bias towards single base substitutions is likely due to polymerase errors during library construction, polymerase errors during the PCR-based amplicon preparation for sequencing, and 454 sequencing errors, all of which would be primarily single base substitutions. The remainder of the bias may reflect the increased mismatch between the mutagenic oligo and the template for codon substitutions with three mutated bases. Still, although codon substitutions with three bp changes may be somewhat disfavored, we observed 95% of the 7749 designed 3-bp change codon substitutions in the 454 sequencing results (Table 1). Since 454 sequencing errors with three bp changes in a codon are likely very rare, we believe most if not all of these 3-base substitutions are present in the library.

### Identification of Adaptive Codon Substitutions in *TEM-1* that Confer Increased Tazobactam Resistance with a Single Amino Acid Substitution

An extensive knowledge of the possible molecular determinants of bacterial resistance to b-lactam antibiotics and ß-lactamase inhibitors would inform the development and implementation of new antibiotics and inhibitors. We identified adaptive codon substitutions in *TEM-1* conferring increased resistance to the ß-lactamase inhibitor tazobactam, which is used clinically in combination with the extended spectrum b-lactam antibiotic piperacillin in the drug Tazocin/Zosyn. We identified these adaptive mutations from library CCM-2– a second comprehensive codon mutagenesis library we constructed that lacked the oligonucleotide-derived bias for G’s in the substituted codon observed in CCM-1 (see Text S1, Figure S2). We subjected CCM-2 to a selection for an increase in resistance to tazobactam. Under the selective conditions, wildtype survived at a frequency of about 10^−3^. Sequencing of 279 colonies revealed 120 unique non-wildtype sequences. Since any particular amino acid substitution is relatively rare in the library, we used the criteria that an amino acid substitution had to be observed twice for us to categorize it as potentially adaptive in nature.

The set of these potentially adaptive substitutions (Table 2) overlapped one (M69L) but not other mutations previously found in alleles that increase tazobactam resistance, most notably R244S and N276D [30]. In addition, we identified 18 new, potentially adaptive amino acid substitutions, the most prevalent of which were 8 different amino acid substitutions at Y105 and the S235T mutation. The Y105 S/D/N and S235T mutations can occur with a single base change and are the most likely to appear naturally. We introduced these four mutations by single base substitution into *TEM-1* and compared the ampicillin, piperacillin, and tazobactam resistance of these alleles to previously known tazobactam resistance alleles (Figure 3). We find that all four provide higher resistance to ampicillin in the presence of tazobactam than R244S and N276D, suggesting that our selection was too strong to identify R244S and N276D. The Y105N, Y105S, and S235T alleles show significant inhibitor resistance for both ampicillin and piperacillin hydrolysis – at or above that of the M69L allele, which is the most resistant allele observed to date for the piperacillin/tazobactam combination [30]. We predict that Y105N, Y105S, and S235T have the potential to emerge in the clinic. Their non-emergence to date, and the fact that they were not identified in previous selections for tazobactam resistance performed on error prone PCR libraries [31] may reflect the fact that the required base substitutions are not as common as the base substitutions for previously identified tazobactam resistance mutations. We speculate that we readily identified these mutations because PFunkel provides a less biased and much more comprehensive library of mutations than error prone PCR.

### PFunkel Mutagenesis using a dsDNA Template

The methods described above require that the gene targeted for mutation is in a phagemid (a plasmid containing the f1 phage origin) and require the production of phage particles from which the dU-ssDNA template is isolated. Although preparation of such a template is straightforward, we sought to expand the method to be applicable to any plasmid and to simplify the protocol by eliminating the need for phage entirely. Phage-less PFunkel (Figure S3, Table S5) achieves this by utilizing a dU-dsDNA plasmid template. After the mutation-containing second strand synthesis, UDG and ExoIII are added to degrade both strands of the dU-dsDNA template. The newly-synthesized, circular ssDNA is then converted to dsDNA using the reverse oligonucleotide. Like PFunkel using a ssDNA template, the reaction can be performed in a single tube using a thermocycler.

We tested PFunkel using a dsDNA template for site-directed mutagenesis and multiple-site mutagenesis. For site-directed mutagenesis (using the c542a mutagenic primer), we obtained 708,000 transformants, and 10 of 10 randomly selected colonies had the desired mutation and no undesired mutations. For multiple-site mutagenesis, we attempted to create four specific mutations at distant sites in the gene. We obtained 445,000 transformants. Four of 10 colonies had all four mutations, the remainder either were wildtype (5 colonies) or had less than 4 mutations (1 colony). We speculate that the apparent lower efficiency of multi-site mutagenesis using a dsDNA template may result from the difficulty in getting all four primers to simultaneously anneal to a dsDNA template (as opposed to a ssDNA template) or difficulty in degrading the dsDNA template. Although we have not tested comprehensive codon mutagenesis using a dsDNA template, we believe it is feasible.

## Discussion

PFunkel offers a very efficient method for site-directed mutagenesis at single or multiple-sites simultaneously. However, the real power of PFunkel lies in the ability to make extensive, user defined libraries of single or multiple mutations. PFunkel can be used for alanine scanning mutagenesis [32] to create all possible alanine substitutions, or a user-defined subset thereof in a single reaction. Comprehensive codon mutagenesis using PFunkel efficiently makes libraries for deep mutational scanning [33] without the need for the costly and time-consuming construction of separate libraries for every codon analyzed. Compared to error prone PCR [34], which can practically access only ∼30% of the possible amino acid substitutions in a gene, comprehensive codon mutagenesis allows effective access to all 100%. For directed evolution studies, the generation of diversity by comprehensive codon mutagenesis will allow access to unique mutational pathways not readily explored by current methods – enabling the identification of unique proteins with improved properties. PFunkel can efficiently create defined mutagenic diversity at multiple sites simultaneously and is thus tailor-made for the creation of computationally designed libraries for subsequent screening or selection strategies.

## Supporting Information

Figure S1 Schematic of the Matlab algorithm for designing the mutagenic oligos for comprehensive codon mutagenesis. For each gene position to be randomized, the algorithm scans through many possible oligos, assigns each a score based on specific guidelines, and then selects the best scoring oligo sequence. Published design criteria [7] along with early experimental data were used to develop the following oligo criteria: a) the oligo length can vary from 27 to 40 bases; b) the mismatched bases must be flanked by ≥12 bases on each side; c) the T_m_ must be ≥ 62°C; d) the GC content must be ≥ 40%; e) oligos with a stable 5′ end and an unstable 3′ end are favored to prevent non-specific annealing and extension; and f) oligos with polynucleotide repeats, hairpin structures, and a propensity for dimerization are penalized. Each oligo is designed to replace a different codon in the *bla* gene with a random sequence (NNN). The script can be easily modified for designing other types of libraries.
(TIF)

Figure S2 Distribution of codon frequencies. Distribution of the frequency of the type of (a) codon substitutions and (b) codons substituted into the comprehensive codon mutagenesis library CCM-1. The frequency is normalized to that expected if all codon substitutions occurred with equal frequency. The codon substitutions are color coded as to the number of G’s in the substituted codon. *TEM-1* lacks three codons (TAG, TGA, AGG) so those codons are not included in the codons substituted into.
(TIF)

Figure S3 Schematic of PFunkel using a dsDNA template. The chief differences from the protocol of Figure 1 are the use of a dsDNA template instead of a ssDNA template and the degradation of the dU-containing template before the third strand synthesis.
(TIF)

Table S1 Reaction conditions for PFunkel using a ssDNA template.
(DOC)

Table S2 Results of additional testing of PFunkel site-directed mutagenesis and multi-site mutagenesis using a single-stranded DNA template.
(DOC)

Table S3 Mutations in 10 clones of the naïve multi-site library.
(DOC)

Table S4 Percent of bases in mutated codons in the comprehensive codon mutagenesis library CCM-1.
(DOC)

Table S5 Reaction conditions for PFunkel using a dsDNA template.
(DOC)

Table S6 Ampicillin MIC values for selected alleles.
(DOC)

Table S7 Piperacillin MIC values for selected alleles.
(DOC)

Text S1 454 GS FLX high-throughput sequencing analysis of the comprehensive codon substitution library; Construction and characterization of comprehensive codon mutagenesis library CCM-2; and PFunkel error rate.
(DOC)
