# Supplementary figures and images for: PFunkel: Efficient, Expansive, User-Defined Mutagenesis
Source: PLoS One. 2012 Dec 17;7(12):e52031. doi: 10.1371/journal.pone.0052031 (PMC3524131; doi:10.1371/journal.pone.0052031)

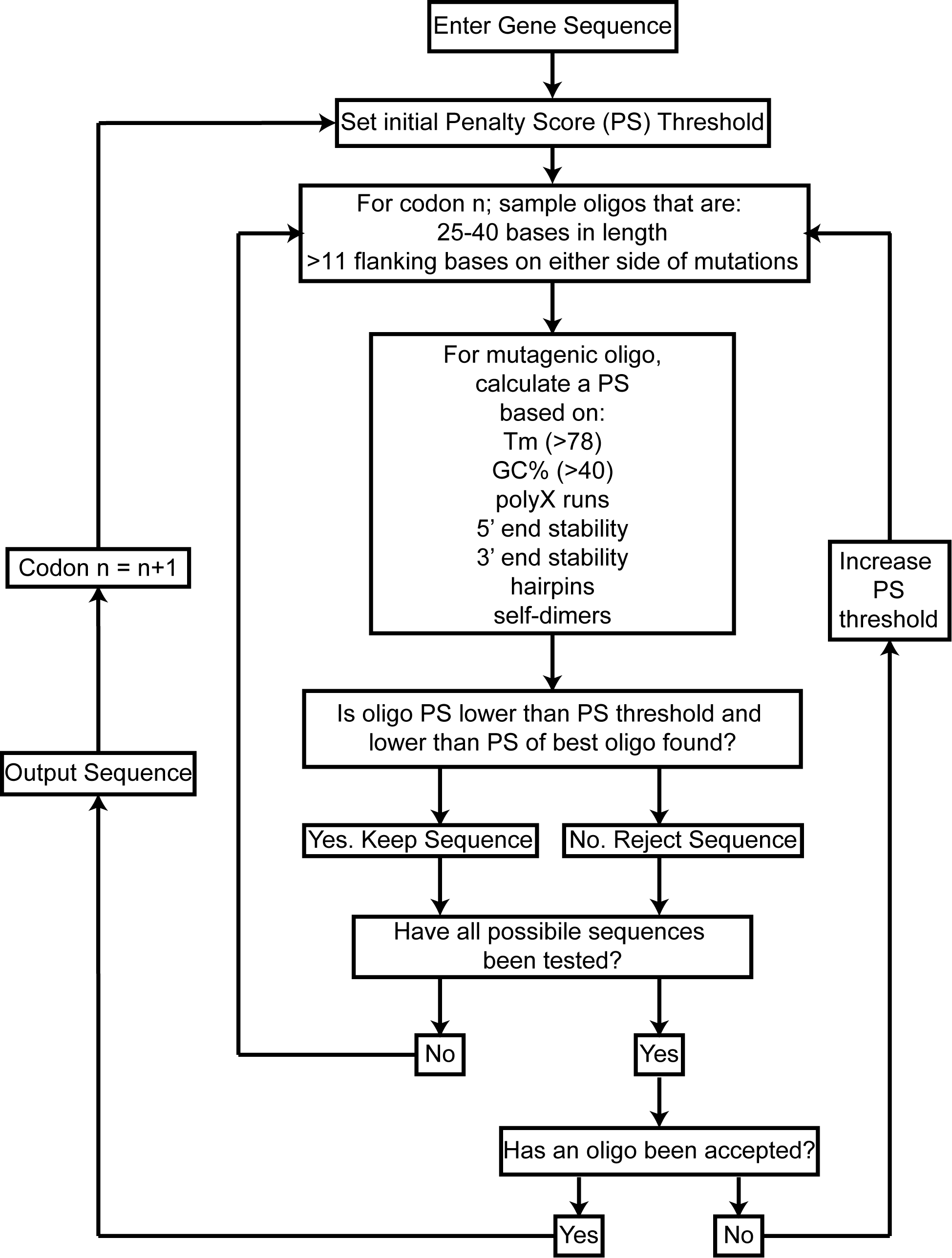

Supplement: Figure S1 — Schematic of the Matlab algorithm for designing the mutagenic oligos for comprehensive codon mutagenesis. For each gene position to be randomized, the algorithm scans through many possible oligos, assigns each a score based on specific guidelines, and then selects the best scoring oligo sequence. Published design criteria [7] along with early experimental data were used to develop the following oligo criteria: a) the oligo length can vary from 27 to 40 bases; b) the mismatched bases must be flanked by ≥12 bases on each side; c) the Tm must be ≥ 62°C; d) the GC content must be ≥ 40%; e) oligos with a stable 5′ end and an unstable 3′ end are favored to prevent non-specific annealing and extension; and f) oligos with polynucleotide repeats, hairpin structures, and a propensity for dimerization are penalized. Each oligo is designed to replace a different codon in the bla gene with a random sequence (NNN). The script can be easily modified for designing other types of libraries. (TIF) [file pone.0052031.s001.tif]

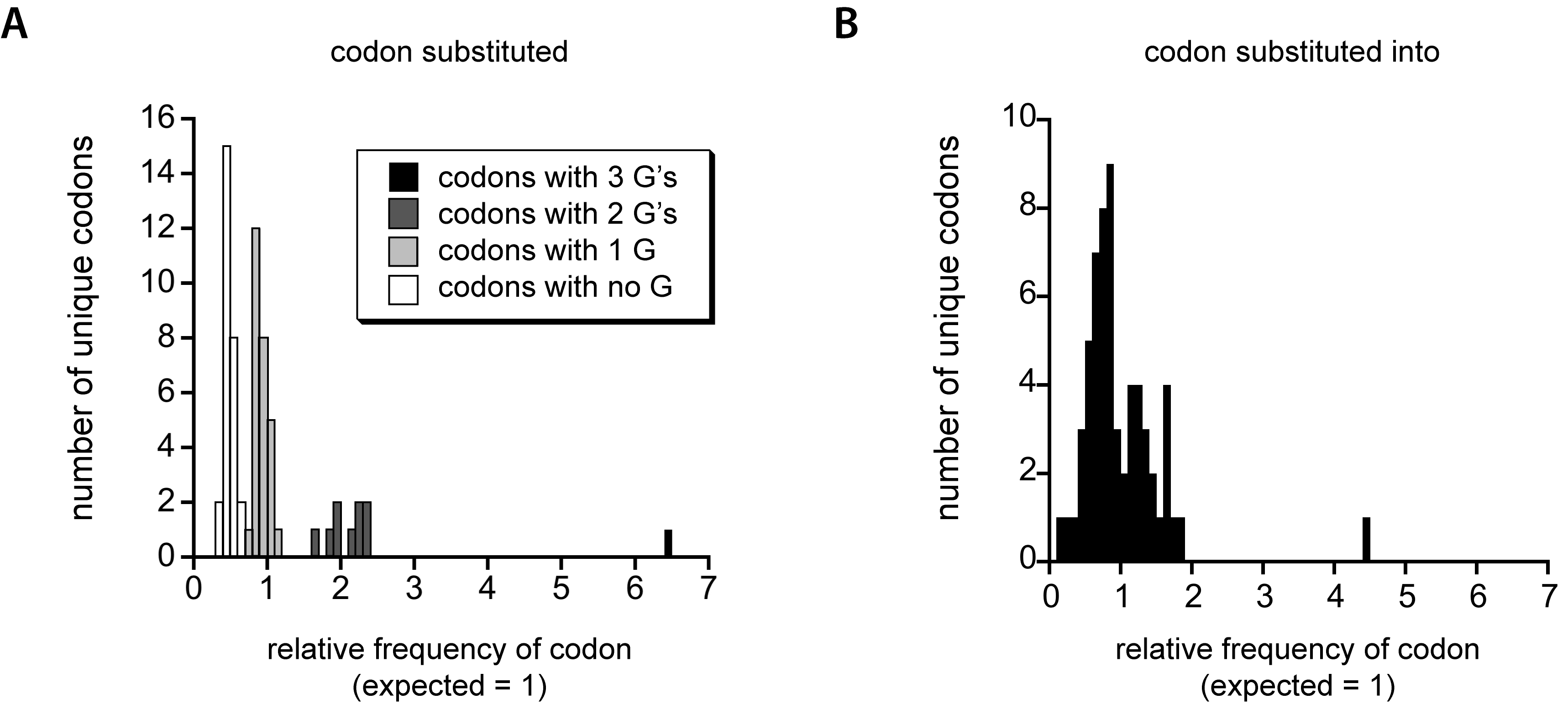

Supplement: Figure S2 — Distribution of codon frequencies. Distribution of the frequency of the type of (a) codon substitutions and (b) codons substituted into the comprehensive codon mutagenesis library CCM-1. The frequency is normalized to that expected if all codon substitutions occurred with equal frequency. The codon substitutions are color coded as to the number of G’s in the substituted codon. TEM-1 lacks three codons (TAG, TGA, AGG) so those codons are not included in the codons substituted into. (TIF) [file pone.0052031.s002.tif]

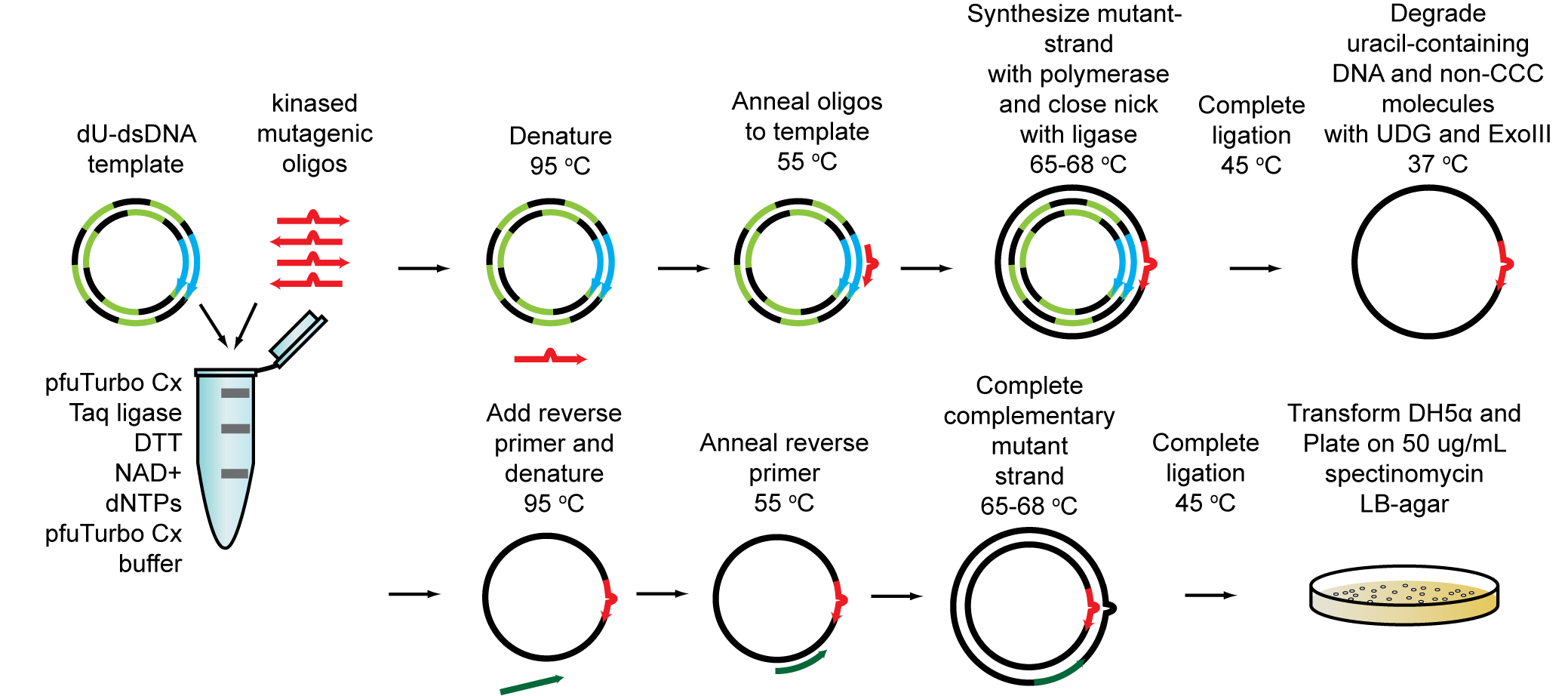

Supplement: Figure S3 — Schematic of PFunkel using a dsDNA template. The chief differences from the protocol of Figure 1 are the use of a dsDNA template instead of a ssDNA template and the degradation of the dU-containing template before the third strand synthesis. (TIF) [file pone.0052031.s003.tif]
