# Supplementary material for: PFunkel: Efficient, Expansive, User-Defined Mutagenesis
Source: PLoS One. 2012 Dec 17;7(12):e52031. doi: 10.1371/journal.pone.0052031 (PMC3524131; doi:10.1371/journal.pone.0052031)
Supplement: Table S1 — Reaction conditions for PFunkel using a ssDNA template. (DOC) [file pone.0052031.s004.doc]

**Table S1.**  Reaction conditions for PFunkel using a ssDNA template.

|  | Site-directed mutagenesis | Multiple-site mutagenesis | | Comprehensive codon mutagenesis | |
| --- | --- | --- | --- | --- | --- |
| Start with | pfuTurbo Cx buffer  2.5 units pfuTurbo Cx polymerase  10 mM DTT  0.5 mM NAD^+^  0.2 mM dNTPs  1 μg (0.75 pmol) of dU-ssDNA  1 μl of 3 μM kinased mutagenic oligo  200 cohesive end units Taq ligase  100 μl total volume | pfuTurbo Cx buffer  1 μg (0.75 pmol) of dU-ssDNA  2 μl of 6 μM kinased mutagenic oligo mixture  77 μl total volume | | pfuTurbo Cx buffer  2.5 units pfuTurbo Cx polymerase  10 mM DTT  0.5 mM NAD^+^  0.2 mM dNTPs  1 μg (0.75 pmol) of dU-ssDNA  1 μl of 0.038 μM kinased mutagenic oligo mix  200 cohesive end units Taq ligase  100 μl total volume | |
| Initial step | none | none | | 95°C for 2 min. | |
| Cycling | 1 cycle of  95 ^o^C for 3 min  55 ^o^C for 90 sec  68 ^o^C for 15 min | 95°C for 3 min,  55°C for 10 min  hold at 55°C.  add  10 mM DTT  0.5 mM NAD^+^,  0.2 mM dNTPs  2.5 units pfuTurbo Cx (previously heat activated)  200 cohesive end units Taq ligase  bringing the total volume to 100 μL  65 ^o^C for 15 min | | 15 cycles of  95°C for 30 sec  55°C for 45 sec  68°C for 6.5 min.  At the 95°C step of cycles 6 and 11, an additional 1 μL of the kinase reaction was added. | |
| Ligation | 45°C for 15 min | | | | |
| add | Add 3.8 pmol of kinased oligo P320 (5:1 molar ratio oligo to template) | | | | |
| Synthesis of second strand containing mutation | one cycle of  95°C for 30 sec  55°C for 45 sec  68°C for 10 min  45°C for 15 min (ligation) | | | | |
| Degradation of template and side-products | Shift to 37°C  Add 10 units of UDG + 30 units of ExoIII; incubate 1 hour at 37°C; 70 ^o^C for 20 min (heat inactivation) | | Shift to 37°C  Add 5 units of UDG + 2 units of ExoIII; incubate 30 min at 37°C; 70 ^o^C for 20 min (heat inactivation) | | Shift to 37°C  Add 10 units of UDG + 30 units of ExoIII; incubate 1 hour at 37°C; 70 ^o^C for 20 min (heat inactivation) |
| Final step | Purify DNA (optional step to increase number of transformants) and transform | | | | |
