# Supplementary material for: PFunkel: Efficient, Expansive, User-Defined Mutagenesis
Source: PLoS One. 2012 Dec 17;7(12):e52031. doi: 10.1371/journal.pone.0052031 (PMC3524131; doi:10.1371/journal.pone.0052031)
Supplement: Table S2 — Results of additional testing of PFunkel site-directed mutagenesis and multi-site mutagenesis using a single-stranded DNA template. (DOC) [file pone.0052031.s005.doc]

**Table S2.** Results of additional testing of PFunkel site-directed mutagenesis and multi-site mutagenesis using a single-stranded DNA template.

| **Type of PFunkel** | **Mutation(s) intended^a^** | **Number of correct sequences^b^** |
| --- | --- | --- |
| Site-directed mutagenesis | M69L | 2 of 2 |
|  | Y105S | 2 of 2 |
|  | Y105D | 2 of 2^c^ |
|  | Y105N | 2 of 2 |
|  | S235T | 2 of 2 |
|  | R244S | 2 of 2 |
|  | N276D | 2 of 2 |
|  | A42G | 2 of 2 |
|  | E104K | 2 of 2^d^ |
|  | M182Q | 2 of 2 |
|  | G238A | 2 of 2 |
| Multi-site mutagenesis | A42G, E104K | 2 of 2 |
|  | A42G, M182Q | 2 of 2 |
|  | A42G, G238A | 2 of 2 |
|  | E104K, M182Q | 2 of 2 |
|  | E104K, G238A | 1 of 2 |
|  | M182Q, G238A | 1 of 2 |
|  | A42G, E104K, M182Q | 1 of 2 |
|  | A42G, E104K, G238A | 1 of 2 |
|  | A42G, M182Q, G238A | 1 of 2 |
|  | E104K, M182Q, G238A | 1 of 2 |
|  | A42G, E104K, M182Q, G238A | 2 of 2 |

^a^ codon change and oligonucleotide used as follows

M69L: atg69ctg (5’- cccgaagaacgttttccaatgctgagcacttttaaa-3’)

Y105S: tac105tcc (5’- tgacttggttgagtcctcaccagtcacaga-3’)

Y105D: tac105gac (5’- tgacttggttgaggactcaccagtcacaga-3’)

Y105N: tac105aac (5’- tgacttggttgagaactcaccagtcacaga-3’)

S235T: tct235act (5’- attgctgataaaactggagccggtgagc-3’)

R244S: cgc244agc (5’- gagcgtgggtctagcggtatcattgca-3’)

N276D: aat276gat (5’- atggatgaacgagatagacagatcgctgaga-3’)

A42G: gca42ggg (5‘- gatcagttgggtgggcgagtgggttac-3’)

E104K: gag104aag (5'- ctcagaatgacttggttaagtactcaccagtcacag -3’)

M182Q: atg182cag (5'- cgtgacaccacgcagcctgcagcaatg -3’)

G238A: ggt238gca (5'- aaatctggagccgcagagcgtgggtct -3’)

^b^The number of clones with all intended mutations out of the total number of clones sequenced.

^c^One clone had an additional, unintended point mutation.

^d^One clone had an additional, unintended mutation found near the desired mutation, presumably resulting from the synthesized mutagenic oligo possessing a misincorporated base.
