# Supplementary material for: PFunkel: Efficient, Expansive, User-Defined Mutagenesis
Source: PLoS One. 2012 Dec 17;7(12):e52031. doi: 10.1371/journal.pone.0052031 (PMC3524131; doi:10.1371/journal.pone.0052031)
Supplement: Table S3 — Mutations in 10 clones of the naïve multi-site library. (DOC) [file pone.0052031.s006.doc]

**Table S3. Mutations in 10 clones of the naïve multi-site library**

| Colony | Ambler Position^a^ | | | | | | | |
| --- | --- | --- | --- | --- | --- | --- | --- | --- |
|  | 42 | | 104 | | 182 | | 238 | |
|  | Codon | Amino acid | Codon | Amino acid | Codon | Amino acid | Codon | Amino acid |
| *TEM-1* | gca | A | gag | E | atg | M | ggt | G |
| 1 | ggc | G | - | - | cag | Q | tgg | W |
| 2 | agc | S | ggg | G | gga | G | - | - |
| 3 | cgg | R | ggg | G | cgg | R | aga | R |
| 4 | tgc | C | ggg | G | agg | R | cgg | R |
| 5 | tgc | C | gcg | A | ggg | G | gtg | V |
| 6 | ggc | G | tgg | W | ccg | P | ctg | L |
| 7 | ggc | G | ggg | G | ttg | L | gac | D |
| 8 | ggt | G | ggg | G | gca | A | atc | I |
| 9 | gcg | A | ggt | G | cat | H | atc | I |
| 10 | - | - | - | - | - | - | tcc | S |

^a^ oligo for position 42: 5’-gatcagttgggtnnncgagtgggttac-3’

oligo for position 104: 5’-gaatgacttggttnnntactcaccagtcac-3’

oligo for position 182: 5’-cgtgacaccacgnnncctgcagcaatg-3’

oligo for position 238: 5’-aaatctggagccnnngagcgtgggtct-3’
