# Supplementary material for: PFunkel: Efficient, Expansive, User-Defined Mutagenesis
Source: PLoS One. 2012 Dec 17;7(12):e52031. doi: 10.1371/journal.pone.0052031 (PMC3524131; doi:10.1371/journal.pone.0052031)
Supplement: Table S4 — Percent of bases in mutated codons in the comprehensive codon mutagenesis library CCM-1. (DOC) [file pone.0052031.s007.doc]

**Table S4.** Percent of bases in mutated codons in the comprehensive codon mutagenesis library CCM-1.

| **Base** | **Expected in an ideal library** | **Sequencing of 90 individual clones of the library** | **454 sequencing of library** | **454 sequencing of *TEM-1* (i.e. sequencing errors)** |
| --- | --- | --- | --- | --- |
| G | 25.00% | 46.25% | 43.26% | 26.85% |
| A | 25.00% | 17.08% | 18.84% | 18.52% |
| T | 25.00% | 18.33% | 18.81% | 24.54% |
| C | 25.00% | 18.33% | 19.09% | 30.09% |
