# Supplementary material for: PFunkel: Efficient, Expansive, User-Defined Mutagenesis
Source: PLoS One. 2012 Dec 17;7(12):e52031. doi: 10.1371/journal.pone.0052031 (PMC3524131; doi:10.1371/journal.pone.0052031)
Supplement: Table S6 — Ampicillin MIC values for selected alleles. (DOC) [file pone.0052031.s009.doc]

**Table S6.** Ampicillin MIC values for selected alleles.

| **Mutation** | **MIC^a^ ampicillin (µg/ml)** | | | | | | | |
| --- | --- | --- | --- | --- | --- | --- | --- | --- |
|  | **– tazobactam** | | | | **+ tazobactam^b^** | | | |
|  | **1** | **2** | **3** | **Median** | **1** | **2** | **3** | **Median** |
| none | 8192 | 8192 | 8192 | 8192 | 16 | 22.6 | 22.6 | 22.6 |
| M69L | 5792 | 8192 | 8192 | 8192 | 512 | 724 | 724 | 724 |
| Y105D | 1448 | 1448 | 1448 | 1448 | 256 | 256 | 362 | 256 |
| Y105N | 4096 | 5792 | 5792 | 5792 | 1448 | 1448 | 1448 | 1448 |
| Y105S | 2896 | 2896 | 2896 | 2896 | 724 | 1024 | 1024 | 1024 |
| S235T | 8192 | 8192 | 8192 | 8192 | 256 | 362 | 512 | 362 |
| R244S | 2896 | 4096 | 4096 | 4096 | 64 | 128 | 90.5 | 90.5 |
| N276D | 8192 | 8192 | 8192 | 8192 | 90.5 | 90.5 | 128 | 90.5 |

^a^ Three replicates and median value. MIC assays performed in √2-fold increments (Mueller Hinton broth-agar, 10^4^ CFU/spot, 37°C for 12 hours).

^b^ tazobactam added to 6 µg/ml
