# Supplementary material for: PFunkel: Efficient, Expansive, User-Defined Mutagenesis
Source: PLoS One. 2012 Dec 17;7(12):e52031. doi: 10.1371/journal.pone.0052031 (PMC3524131; doi:10.1371/journal.pone.0052031)
Supplement: Table S7 — Piperacillin MIC values for selected alleles. (DOC) [file pone.0052031.s010.doc]

**Table S7.** Piperacillin MIC values for selected alleles.

| **Mutation** | **MIC^a^ piperacillin (µg/ml)** | | | | | | | |
| --- | --- | --- | --- | --- | --- | --- | --- | --- |
|  | **– tazobactam** | | | | **+ tazobactam^b^** | | | |
|  | **1** | **2** | **3** | **Median** | **1** | **2** | **3** | **Median** |
| none | 2896 | 2896 | 2896 | 2896 | 1 | 1.4 | 1.4 | 1.4 |
| M69L | 2048 | 2896 | 2896 | 2896 | 22.6 | 32 | 22.6 | 22.6 |
| Y105D | 1.4 | 1.4 | 1.4 | 1.4 | 1 | 1 | 1 | 1 |
| Y105N | 2048 | 2048 | 2048 | 2048 | 32 | 45 | 45 | 45 |
| Y105S | 1024 | 1448 | 1448 | 1448 | 16 | 16 | 16 | 16 |
| S235T | 2896 | 2896 | 2896 | 2896 | 32 | 16 | 22.6 | 22.6 |
| R244S | 1024 | 1448 | 1448 | 1448 | 2 | 2.83 | 2 | 2 |
| N276D | 2896 | 2896 | 2896 | 2896 | 1 | 1.4 | 2 | 1.4 |

^a^ Three replicates and median value. MIC assays performed in √2-fold increments (Mueller Hinton broth-agar, 10^4^ CFU/spot, 37°C for 12 hours).

^b^ tazobactam added to 6 µg/ml
