# Supplementary material for: PFunkel: Efficient, Expansive, User-Defined Mutagenesis
Source: PLoS One. 2012 Dec 17;7(12):e52031. doi: 10.1371/journal.pone.0052031 (PMC3524131; doi:10.1371/journal.pone.0052031)
Supplement: Text S1 — 454 GS FLX high-throughput sequencing analysis of the comprehensive codon substitution library; Construction and characterization of comprehensive codon mutagenesis library CCM-2; and PFunkel error rate. (DOC) [file pone.0052031.s011.doc]

**Text S1**

**454 GS FLX high-throughput sequencing analysis of the comprehensive codon substitution library.**

Barcoded amplicons from the three CCM-1 libraries were created by PCR and pooled. Additionally, barcoded amplicons created from the wildtype *TEM-1* gene were added to the pool as a control for sequencing errors. We obtained 787,488 reads that passed quality filtering, with a median length of 354 bases. A total of 99% of the reads spanned the entire mutated region of the amplicon. The reads of the library DNA displayed a higher frequency of both wildtype (26%) and multiple mutations (17%) at the expense of single codon mutations (57%) as compared to the Sanger sequencing of 90 clones. However, this was determined to be an artifact of the amplicon preparation known as “PCR jumping,” a well-documented occurrence during PCR amplification of highly-identical, heterogeneous template sequences in which chimera PCR products are produced [1,2]. We confirmed this was the case by Sanger sequencing of 28 individual PCR amplicons of which 36% had no mutations, 50% had one codon mutation, and 14% had multiple codon mutations (Table 1). This closely matched the proportions in the 454 sequencing. The sequencing of wild-type *TEM-1* indicated that the sequencing error rate (0.035 codon substitutions per read) was much less than the frequency of codon substitutions observed in the reads of the library DNA (0.94 per read). We conclude that 96% of the codon substitutions observed in the 454 sequencing reads are present in the library, with the remainder being sequencing errors. Of the codon substitutions present in the library, ≤3% are present in library members with multiple mutations (based on Sanger sequencing).

In the 454 sequencing reads of the library we observed 97.0% of the 18,081 intended codon substitutions at least once. In the worst-case scenario in which sequencing errors are assigned to mutations with the lowest numbers of occurrences, 84.8% of the 18,081 possible mutants are present in the library. If sequencing errors are evenly distributed across all codon substitutions, 96.4% are present. In the best-case scenario in which errors are assigned to mutants that are highly represented, 96.8% of the 18,081 mutants are present. We believe that the true coverage of the library lies between 96.4% and 97% and likely closer to 97%, since 454 sequencing is known to exhibit sequence-dependent common errors. Among the 72 sequencing errors in the reads of wild-type *TEM-1*, one particular substitution appeared five times and five codon substitutions appeared twice. More extensive sequencing of wild-type *TEM-1* would be necessary to accurately determine the frequency at which each of the 18,081 possible codon substitutions appear because of sequencing error, and thus the true frequency of each codon substitution in the library.

Both the Sanger and the 454 sequencing indicate that G’s are present in mutated codons 2.3 times more frequently than any one of the other three bases (Table S4). The high frequency of G’s is also apparent in the sequences of naïve members of the multi-site mutagenesis library (Table S3), which used four specific primers from the set of 287. The distribution of the frequency of the substituted codons strongly reflects this bias (Figure S2A) whereas the distribution of the frequency of codons substituted into does not (Figure S2B). Since *TEM-1* has roughly an equal frequency of all bases, we conclude that this bias results from a 2.3-fold bias for incorporation of G’s during the synthesis of the machine-mixed degenerate oligonucleotides. This bias contributed to the underrepresentation of certain mutations, as the frequency of G’s in codon substitutions not observed in the 454 sequencing was 0.068.

**Construction and characterization of comprehensive codon mutagenesis library CCM-2.**

To confirm that the bias for G’s resulted from their overrepresentation in the mutagenic oligos, we constructed a second set of three libraries (CCM-2) using a second set of degenerate mutagenic oligos that were synthesized using a hand-mixed ratio of bases (instead of machine mixed). The three libraries were transformed into NEB 5-alpha F’I^q^ cells, which contain the lacI^q^ repressor to better repress expression to avoid any bias when propagating the library. Sequencing of 30 members of each library revealed 8.9% wildtype, 83.1% single codon substitutions in the targeted region, 1.1% with a single mutation outside the targeted region, and 6.7% multiple mutations (3 of 6 had two- mutations in the targeted region; 3 of 6 had one codon substitution in the targeted region and the second mutation in a non-targeted region). The frequency of bases substituted in the designed mutations of CCM-2 (27.5%:26.6%:23.0%:23.0% for G:A:C:T) was much more even than in CCM-1. The ratio of 1-base:2-base:3-base substitutions in the targeted region was 27.7%:42.2%:30.1%.

**PFunkel error rate**

PfuTurbo Cx hotstart DNA polymerase has a published error rate of 1.3 x 10^-6^ in a PCR reaction using a double-stranded template [3]. For site-directed and multi-site mutagenesis using a single-stranded template we observed three unintended mutations outside the region of the mutagenic oligo in 77 sequencing reactions of the 861 bp *TEM-1* gene, which corresponds to an error rate of 4.5 x 10^-5^. For the comprehensive codon mutagenesis, we observed 6 mutations outside the target region in CCM-1 and CCM-2, which corresponds to an error rate of 5.8 x 10^-5^. These error rates are 35- and 45-fold higher than PfuTurbo Cx hotstart DNA polymerase’s error rate. We speculate that the elevated error rate results from deviations from the recommended PfuTurbo Cx reaction buffer and/or degradation of the ssDNA template at 95°C. All the observed unintended mutations can be explained by cytosine deamination (~2 x 10^-7^ events/sec at 95°C in ssDNA [4]) leading to G:C->A:T transitions or depurination (~4 x 10^-7^ events/sec at 95°C in ssDNA [5,6]) which can lead to various mutations. When ssDNA is used as the template, we speculate that the 95°C incubation step is not essential for PFunkel and that elimination of this step would lower the error rate.

**References**

1. Holland MM, McQuillan MR, O'Hanlon KA (2011) Second generation sequencing allows for mtdna mixture deconvolution and high resolution detection of heteroplasmy. Croat Med J 52: 299-313.

2. Meyerhans A, Vartanian JP, Wain-Hobson S (1990) DNA recombination during PCR. Nucleic Acids Res 18: 1687-1691.

3. PfuTurbo Cx hotstart DNA polymerase Instruction Manual, (2009) Catalog #600410, Revision A.01. Agilent Technologies.

4. Lindahl T, Nyberg B (1974) Heat-Induced deamination of cytosine residues in deoxyribonucleic acid. Biochemistry 13: 3405-3410.

5. Lindahl T, Nyberg B (1972) Rate of depurination of native deoxyribonucleic acid. Biochemistry 11: 3610-3618.

6. André P, Kim A, Khrapko K, Thilly WG (1997) Fidelity and mutational spectrum of pfu DNA polymerase on a human mitochondrial DNA sequence. Genome Res 7: 843-852.

7. QuikChange Site-Directed Mutagenesis Kit, Instruction Manual, Catalog #200518, Revision #B.01, Stratagene.
